# Xylose-Oligosaccharide Alleviating Type 2 Diabetes in Mice via Reducing Blood Glucose, Oxidative Stress, Inflammation and Regulating Intestinal Microbiota

**DOI:** 10.3390/foods14122093

**Published:** 2025-06-13

**Authors:** Xiangfei Li, Xiaofeng Xia, Zifan Cai, Xinyi Pang, Jing Sun, Yingjian Lu

**Affiliations:** College of Food Science and Engineering, Nanjing University of Finance and Economics, Nanjing 210023, China; xiangfeili@nufe.edu.cn (X.L.); 15995644253@163.com (X.X.); mycorftalter@foxmail.com (Z.C.); ; pangxinyi@nufe.edu.cn (X.P.); jingsun@nufe.edu.cn (J.S.)

**Keywords:** xylo-oligosaccharide, diabetes, insulin resistance, oxidative stress, inflammatory, gut microbiota

## Abstract

Type 2 diabetes, a widespread metabolic disorder, is characterized by hyperglycemia and insulin resistance. Xylose-oligosaccharide, a functional oligosaccharide, has shown potential in mitigating hyperglycemia. This study established a type 2 diabetes mouse model via a high-fat diet and streptozotocin administration to investigate the effects of two xylose-oligosaccharide doses (20 and 60 mg/kg/day). Both doses were observed to regulate lipid metabolism, decrease inflammation, and alleviate damage to the liver, kidneys, and islets. Additionally, xylose-oligosaccharide corrected diabetes-induced intestinal microbiota imbalances by increasing Bacteroides and Proteobacteria while decreasing Firmicutes. Notably, the 60 mg/kg/day dose was more effective in enhancing glucose tolerance and reducing insulin resistance compared to the 20 mg/kg/day dose. These results suggested that xylose-oligosaccharide had hypoglycemic effects, reduced insulin resistance and oxidative stress, possessed anti-inflammatory properties, and modulated intestinal microbiota. Thus, xylose-oligosaccharide shows promise as a functional food for managing type 2 diabetes.

## 1. Introduction

Diabetes is a persistent metabolic disorder that has seen its prevalence more than double globally over the past two decades, posing significant risks to public health and the global economy [1]. Type 2 diabetes (T2D), which constitutes over 90% of diabetes cases, is marked by hyperglycemia, hyperlipidemia, relative insulin deficiency, and insulin resistance [2]. These conditions can lead to severe complications such as cardiovascular disease, stroke, and diabetic retinopathy. Obesity, commonly associated with T2D, results from insulin resistance and relative insulin deficiency, causing fat accumulation. Adipose tissue not only stores fat but also secretes various inflammatory factors that contribute to physiological and pathological processes [3]. Chronic inflammation has been identified as a key factor in T2D progression, with studies highlighting inflammation as a pathway to diabetic complications [4,5]. Hyperglycemia-induced oxidative stress elevates pro-inflammatory factor levels, and macrophages secrete inflammatory factors, triggering inflammation [6,7]. In T2D patients, elevated levels of central inflammatory cytokines such as interleukin (IL)-1, IL-6, and tumor necrosis factor-α (TNF-α) were observed [8,9]. The intestinal microbiota significantly influences host metabolism, and its imbalance is closely linked to T2D [10,11,12].

Current T2D treatments primarily rely on medications, which are associated with side effects such as vitamin B12 deficiency, gastrointestinal disturbances, and hepatotoxicity [13]. In contrast, dietary interventions have emerged as a promising strategy for T2D prevention and management, with low-calorie sugars and sugar alcohols garnering attention as safer alternatives to conventional drugs [14]. For instance, after treatment with agar oligosaccharides and neo-agar oligosaccharides with an even number of sugar units, colitis and colonic microbiota dysregulation in T2D mice can be improved, thereby alleviating oxidative stress, inflammation, and associated hyperglycemia, insulin resistance, lipid accumulation, and obesity in high-fat-diet-induced T2D [15,16,17]. In addition, most oligosaccharides such as fructo-oligosaccharides, galacto-oligosaccharides, and trehalose have been shown to exert prebiotic effects. These prebiotics can directly influence blood glucose levels, improve gut microbial composition, and support blood glucose control in patients with T2D. These examples demonstrate the potential of oligosaccharides as personalized dietary interventions for optimizing diabetes management and improving overall health outcomes [18,19,20].

Xylose-oligosaccharide, an oligosaccharide composed of D-xylose units linked via β-1,4-glycosidic bonds, penetrates cell membranes, participates in glucose metabolism without raising blood glucose levels, and stimulates insulin production, making it beneficial for energy supplementation and glucose metabolism improvement. Studies have demonstrated that dietary supplementation with both 0.5 g/kg and 1 g/kg xylose-oligosaccharide significantly alleviated (*p* < 0.05) the *Eimeria*-induced upregulation of Claudin 1 expression. Regarding nutrient transporters, 0.5 g/kg xylose-oligosaccharide supplementation downregulated (*p* < 0.01) the expression of GLUT2 and GLUT5, thereby enhancing growth performance in broilers [21]. Dietary D-xylose has also been found to promote intestinal health by inducing bacteriophage production to inhibit *E. coli* [22].

The aim of this study is to systematically investigate the alleviating effect of xylose-oligosaccharides on T2D and its potential mechanisms. We established a T2D mouse model using a high-fat diet combined with streptozotocin (STZ). We administered low-dose and high-dose xylose-oligosaccharides by gavage to evaluate their effects on glucose regulation, lipid metabolism, oxidative stress, inflammatory response, and key organ pathological damage. Additionally, we analyze its regulation of intestinal microbiota composition, particularly the abundance of key phyla (e.g., Bacteroidetes, Firmicutes). By comparing the effects of different doses, this study aims to elucidate how xylose-oligosaccharides improve T2D-related metabolic abnormalities and intestinal dysbiosis and evaluate their potential as functional foods or therapeutic agents for T2D management.

## 2. Materials and Methods

### 2.1. Chemicals and Reagents

Xylose-oligosaccharide (Yuanye, Shanghai, China); mice standard chow diet and high-fat diet (60% fat, HFK Bioscience, Beijing, China); STZ and metformin (Sigma Chemical Co., St. Louis, MO, USA); blood glucose monitor and test strips (HMD Biomedical Inc., Taiwan, China); insulin (Novo Nordisk, Bagsværd, Denmark).

### 2.2. Mice and Intervention Protocol

All animal experimental procedures were approved by the Animal Ethics Committee of Nanjing Agricultural University, China (SYXK 2017-0007). Fifty specific-pathogen-free, four-week-old male C57BL/6J mice were obtained from Beijing Vital River Laboratory Animal Technology Co., Ltd. (SCXK(JING) 2016–0011, Beijing, China). The mice were housed under controlled conditions: a constant temperature (22 °C ± 2 °C), humidity (55% ± 5%), a 12 h light–dark cycle, and an air exchange rate of 15 times/h.

During the entire study, all mice had free access to food and water ad libitum. The standard chow diet contained 10% fat, 20% protein, and 70% carbohydrates, while the high-fat diet was composed of 60% fat, 20% protein, and 20% carbohydrates. Diet batches were sterilized by gamma irradiation and stored at −20 °C to prevent oxidation. Following a three-week acclimatization period, mice were randomly assigned to five groups (n = 10 per group):

Control (CON) group: standard chow diet, 0.4 mL ultrapure water intragastrically.

Diabetic model (DM) group: high-fat diet, 0.4 mL ultrapure water intragastrically.

Positive control (PC) group: high-fat diet, 0.4 mL 300 mg/kg body weight (bw) metformin intragastrically.

Low dose of xylose-oligosaccharide intragastric administration (LXY) group: high-fat diet, 0.4 mL 20 mg/kg bw xylose-oligosaccharide intragastrically.

High dose of xylose-oligosaccharide intragastric administration (HXY) group: high-fat diet, 0.4 mL 60 mg/kg bw xylose-oligosaccharide intragastrically.

**Figure 1:**
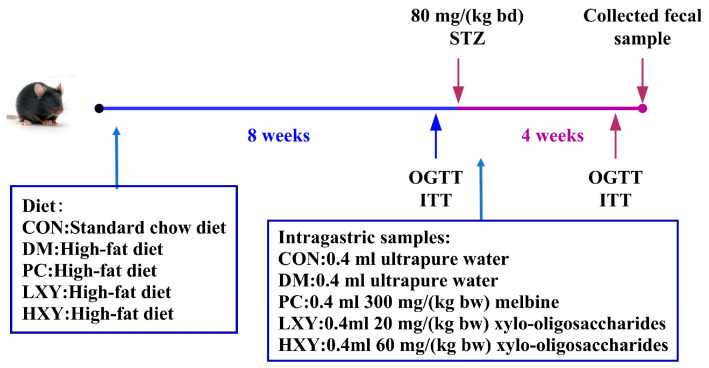
Diet and intragastric samples of mice in different groups.

The dose of xylose-oligosaccharides used was determined based on previous toxicity studies by other researchers. After an 8-week dietary intervention, all mice were fasted for 12 h and received intraperitoneal injections of STZ (or citrate buffer for the CON). The study continued for an additional 4 weeks to evaluate the effects of xylose-oligosaccharides on diabetic parameters. Mice fed a high-fat diet received freshly prepared STZ dissolved in sterile citrate buffer (pH 4.5) at a dose of 80 mg/kg bw to induce type 2 diabetes, while mice on a normal diet were injected with an equal volume of citrate buffer (pH 4.5) alone (Figure 1). At the end of week 12, mice were euthanized by cervical dislocation. Epididymal white adipose tissue and hepatic, pancreatic, and renal tissues were rinsed with cold 0.85% saline solution to remove surface blood, weighed, and subsequently fixed in 10% formaldehyde solution for histological preservation and analysis. Blood samples were collected via orbital puncture and centrifuged at 3000× *g* for 10 min to isolate serum for further biochemical assessments. All collected samples were stored at −80 °C for further analysis.

### 2.3. Biochemical Analysis

Fasting blood glucose (FBG) levels were measured weekly using a blood glucose monitor. At week 12, feces were collected and stored at −80 °C. An oral glucose tolerance test (OGTT) was conducted at week 12 after 12 h of fasting, recording blood glucose levels at 0, 30, 60, and 120 min post-glucose administration (2 g/kg bw). Three days before the study concluded, an insulin tolerance test (ITT) was performed after 5 h of fasting, with blood glucose levels recorded at 0, 30, 60, 90, and 120 min post-insulin administration (0.75 U/kg bw).

After blood collection, samples were centrifuged at 3000× *g* and 4 °C for 10 min to extract serum. Using a tissue grinder (Wheaton, Kimble, Millville, NJ, USA), part of the liver tissue was mixed with PBS (0.25 g mL^−1^; White Shark Biotechnology Co., Ltd., Hefei, China), followed by centrifugation at 2000 g for 15 min. The supernatant was collected and prepared as a 20% liver homogenate for subsequent hepatic biochemical assays. Triglyceride (TG), low-density lipoprotein cholesterol (LDL-C), high-density lipoprotein cholesterol (HDL-C), and total cholesterol (TCHO) levels were measured using commercial kits (Nanjing Jiancheng Biology Engineering Institute, Nanjing, China). Serum levels of interleukin-1β (IL-1β), interleukin-6 (IL-6), interleukin-10 (IL-10), and tumor necrosis factor-α (TNF-α) were determined using enzyme-linked immunosorbent assay (ELISA) kits from the same supplier. Hepatic superoxide dismutase (SOD), glutathione (GSH), glutathione peroxidase (GSH-PX), and malondialdehyde (MDA) levels were also measured using commercial kits ((Nanjing Jiancheng Biology Engineering Institute, Nanjing, China).

### 2.4. Histopathological Examination

Histopathological examinations of adipose, pancreatic, and hepatic tissues were conducted by Shanghai Erwan Biotechnology Co., Ltd. (Shanghai, China).. The embedded specimens were sectioned into 5-μm-thick paraffin sections using a microtome (Leica Biosystems, Nussloch, Germany). Following baking at 60 °C for 1 h, the sections were sequentially deparaffinized with xylene I (China National Pharmaceutical Group Chemical Reagent Co., Ltd., Shanghai, China) and xylene II (same supplier) for 10 min each, followed by rehydration through gradient alcohol solutions (from 100% to 70% ethanol). Nuclei were then stained with hematoxylin for 3 min, differentiated in a hydrochloric acid–ethanol solution for 1–2 s to enhance contrast, and rinsed. The samples were counterstained with eosin for 1 min, dehydrated through a graded ethanol series (70% to 100%), and cleared with xylene I and xylene II for 5 min each to remove ethanol and facilitate mounting. Finally, the sections were sealed with neutral balsam (China National Pharmaceutical Group Chemical Reagent Co., Ltd., Shanghai, China). After staining, tissue cell nuclei exhibited a distinct blue coloration, while the cytoplasm appeared pink, enabling clear histological differentiation under light microscopy [23].

### 2.5. Fecal Microbiota Analysis by Metagenome

Gut microbiota analysis was performed by the Beijing Genomics Institute using high-throughput sequencing technology. Sequence data were processed using QIIME2 2024.2 software, with operational taxonomic units (OTUs) clustered at 97% similarity.

### 2.6. Statistical Analysis

All tests were conducted in triplicate. Statistical analyses of the data were performed using SPSS 16.0 software with a one-way analysis of variance (ANOVA) followed by the Tukey test. Data are presented as mean ± SEM. GraphPad Prism 5 was used for visualizing experimental results. A probability level (*p*) of less than 0.05 was considered statistically significant.

## 3. Results

### 3.1. Effects of Xylose-Oligosaccharide on FBG

**Figure 2:**
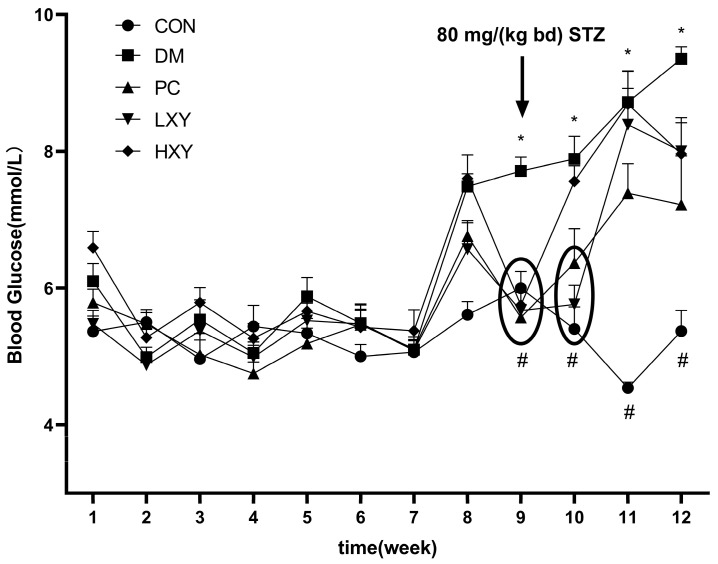
FBG during the experiment period. Values are mean ± SEM for ten mice in each group. * *p* < 0.05 vs. CON group and # *p* < 0.05 vs. DM group.

The effects of xylose-oligosaccharide on diabetic mice were assessed by recording FBG (Figure 2). The FBG levels in the DM group were significantly higher than those in the CON group at weeks 9, 10, 11, and 12. Conversely, the FBG levels in the LXY and HXY groups were significantly lower than those in the DM group at weeks 9 and 10 (Figure 2, *p* < 0.05).

### 3.2. Effect of Xylose-Oligosaccharide on OGTTs and ITTs

**Figure 3:**
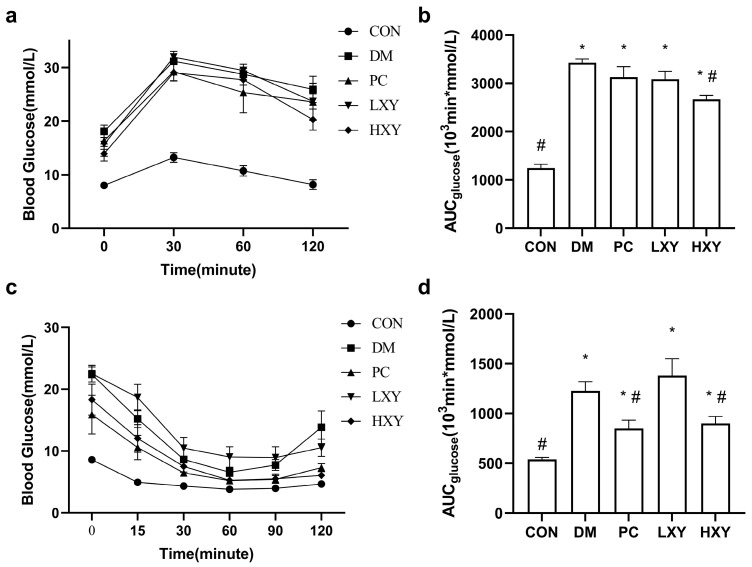
Effect of xylose-oligosaccharide on glucose tolerance and insulin tolerance in DM mice. (**a**) Oral glucose tolerance test. (**b**) AUC of glucose over 120 min in OGTT. (**c**) Insulin tolerance test. (**d**) AUC of glucose over 120 min in ITT. Values are mean ± SEM for ten mice in each group. * *p* < 0.05 vs. CON group and # *p* < 0.05 vs. DM group.

The OGTT was used to evaluate glucose tolerance in diabetic mice. As shown in Figure 3a, the blood glucose levels in the DM group were significantly higher than those in the CON group, while these levels were reduced in the HXY group (*p* < 0.05). After 120 min, the blood glucose levels in the HXY group were significantly lower than those in the PC group, suggesting that high-dose xylose-oligosaccharide may enhance glucose tolerance more effectively than metformin. These findings are corroborated by Figure 3b, which showed that the blood glucose levels in the DM group were significantly higher than those in the CON group, but this increase was alleviated in the HXY group (*p* < 0.05).

The ITT was conducted to assess insulin sensitivity. Following insulin injection, both the PC and HXY groups exhibited better responses to insulin compared to the DM group (Figure 3c). The area under the curve (AUC) for glucose in the PC and HXY groups was significantly lower (30.7% and 26.6%, respectively) than in the DM group (*p* < 0.05, Figure 3d). This indicated that high-dose xylose-oligosaccharide can improve insulin sensitivity and reduce insulin resistance in T2D mice.

### 3.3. Effect of Xylose-Oligosaccharide on Serum Lipid

**Figure 4:**
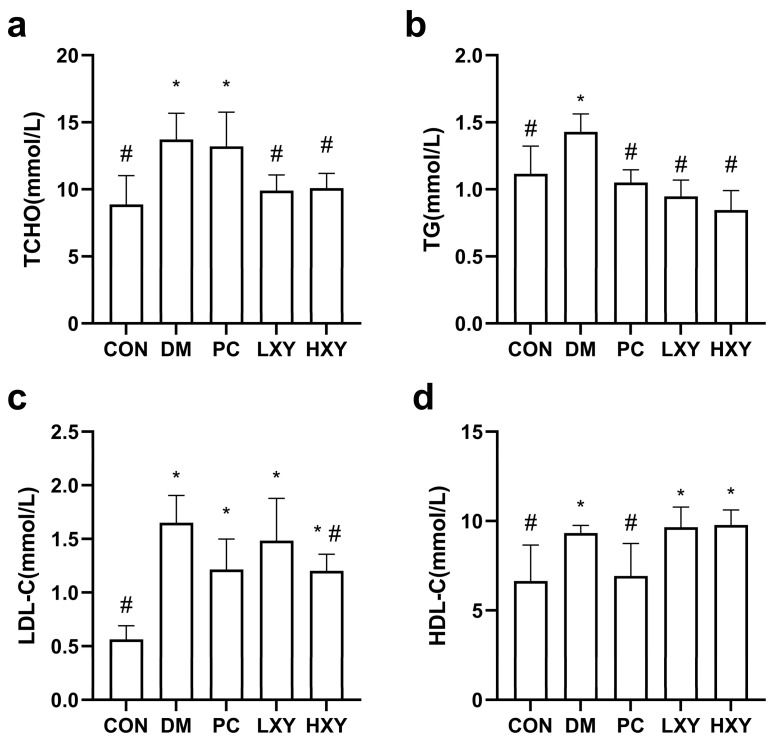
Effect of xylose-oligosaccharide on serum lipid levels in mice. (**a**) TG. (**b**) LDL-C. (**c**) HDL-C (**d**) TCHO. Values are mean ± SEM for ten mice in each group. * *p* < 0.05 vs. CON group and # *p* < 0.05 vs. DM group.

The levels of total cholesterol (TCHO), triglycerides (TG), low-density lipoprotein cholesterol (LDL-C), and high-density lipoprotein cholesterol (HDL-C) in the DM group were 54.62%, 28.05%, 193.43%, and 40.43% higher, respectively, compared to the CON group. The LXY group showed significantly lower levels of TCHO and TG compared to the DM group, although there were no significant differences in LDL-C and HDL-C. The HXY group exhibited even greater improvements, with decreases of 35.84%, 68.81%, and 34.97% in TCHO, TG, and HDL-C, respectively (Figure 4, *p* < 0.05).

### 3.4. Effect of Xylose-Oligosaccharide on Inflammatory Factors and Antioxidant Parameters

**Figure 5:**
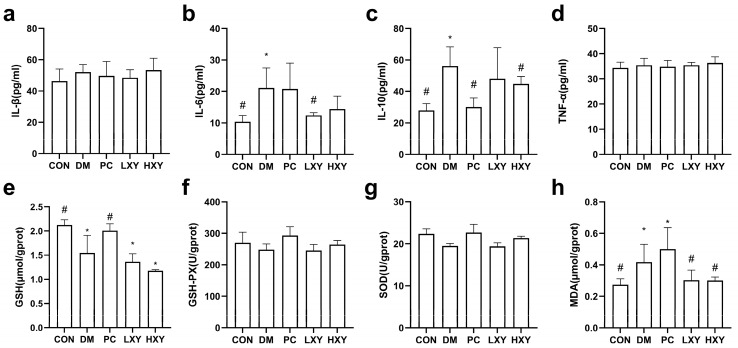
Effect of xylose-oligosaccharide on serum inflammatory factors and antioxidant parameters. (**a**) IL-1β. (**b**) IL-6. (**c**) IL-10. (**d**) TNF-α. (**e**) GSH. (**f**) GSH-PX. (**g**) SOD. (**h**) MDA. Values are mean ± SEM for ten mice in each group. * *p* < 0.05 vs. CON group and # *p* < 0.05 vs. DM group.

The levels of inflammatory factors such as IL-1β, IL-6, IL-10, and TNF-α, as well as oxidative stress markers like GSH, GSH-PX, SOD, and MDA, were measured. Inflammatory factors indicate the extent of inflammatory damage. In type 2 diabetic mice, IL-6 and IL-10 are key mediators of inflammatory responses. The IL-6 level in the LXY group decreased significantly by 70.58%, while the IL-10 level in the HXY group decreased by 25.10%, returning to levels similar to the CON group (Figure 5b,c, *p* < 0.05).

The MDA level in the DM group was significantly higher than in the CON group. Both the LXY and HXY groups showed significant reductions in MDA levels by 38.14% and 39.10%, respectively, compared to the DM group (Figure 5h, *p* < 0.05). However, there were no significant differences in the levels of GSH, GSH-PX, and SOD between the LXY, HXY, and DM groups (Figure 5e,f,g, *p* < 0.05).

### 3.5. Effect of Xylose-Oligosaccharide on Histopathology of the Epididymal White Adipose Tissue, Liver Tissue, and Pancreas Tissue

**Figure 6:**
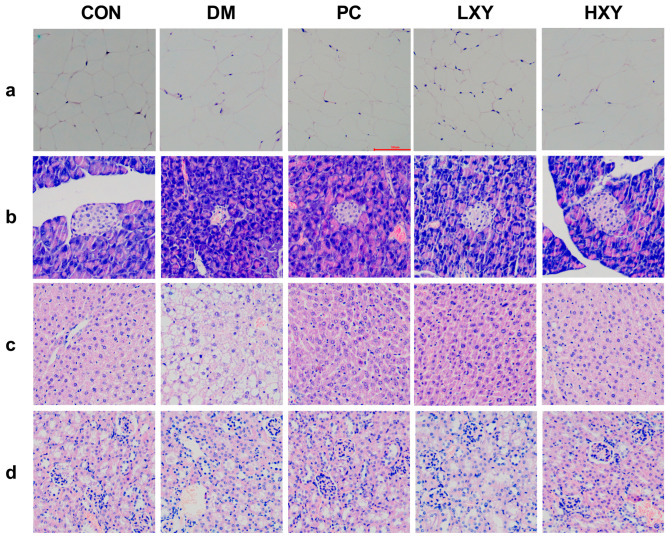
Effect of xylose-oligosaccharide on the epididymal white adipose tissue. (**a**) White adipose tissue, (**b**) liver tissue, (**c**) pancreas tissue, and (**d**) kidney tissue liver tissue (Magnification, 200×).

To directly investigate the effects of xylose-oligosaccharide on major organs in mice, histopathological examinations were performed on the epididymal white adipose tissue, liver tissue, pancreatic tissue, and kidney tissue of the mice. In the CON group, adipocytes were neatly arranged and densely packed, whereas in the DM group, adipocytes were larger and irregularly shaped. The adipocytes in the PC, LXY, and HXY groups were smaller than those in the DM group (Figure 6a).

H&E staining revealed increased numbers and sizes of fat vacuoles in the liver tissue of the DM group. In contrast, mice treated with metformin and xylose-oligosaccharide showed significant reductions in fat vacuoles (Figure 6b). Pancreatic histopathology showed that islet cells in the CON group were round with clear, intact borders, while islets in the DM group were irregular, smaller, and fewer in number (Figure 6c). Treatment with metformin or xylose-oligosaccharide restored the morphology of islets. Additionally, glomerular volume increased, and renal tubule organization was disrupted in the DM group compared to the CON group, but these pathological changes were less severe in the PC, LXY, and HXY groups (Figure 6d). These data suggested that xylose-oligosaccharide may help prevent tissue disorders in T2D mice.

### 3.6. Effect of Xylose-Oligosaccharide on Gut Microbiota

**Figure 7:**
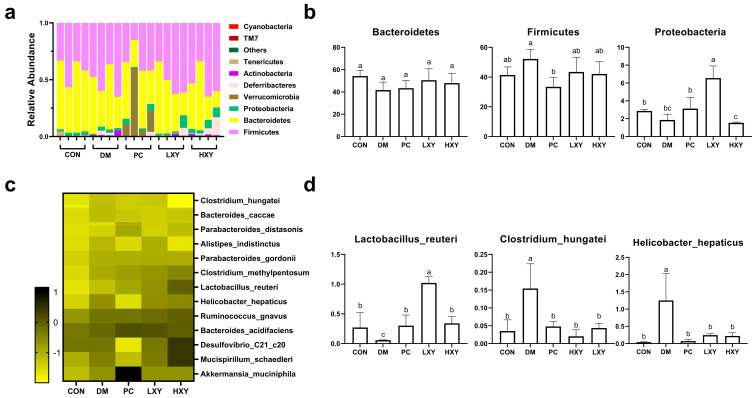
Effect of xylose-oligosaccharide on gut microbiota in high-fat and STZ induced mice. (**a**) Relative abundance of five groups at the phylum level; (**b**) relative abundance of major phyla in the five groups; (**c**) heatmap of 13 most predictive phylotypes; (**d**) relative abundance of genera in the five groups. Values are expressed as mean ± SEM (n = 5). (* *p* < 0.05).

To elucidate the impact of xylose-oligosaccharide on the gut microbiota of diabetic mice, a 16S rRNA metagenomic analysis was performed. At the phylum level, Bacteroidetes, Firmicutes, and Proteobacteria constituted 90% of the total microbiota (Figure 7a). In the DM group, the abundance of Bacteroidetes and Proteobacteria decreased significantly, while Firmicutes increased markedly compared to the CON group. Xylose-oligosaccharide treatment restored the abundance of Bacteroidetes in the LXY and HXY groups, even exceeding the levels observed in the PC group. Conversely, the abundance of Firmicutes decreased significantly in the PC, LXY, and HXY groups relative to the DM group. Additionally, the concentration of Proteobacteria in the LXY and HXY groups was effectively restored to levels similar to those of the CON group (Figure 7b).

At the genus level, xylose-oligosaccharide treatment notably influenced the relative abundance of specific genera within the fecal microbiota of diabetic mice. Significant alterations were observed in the relative abundance of *Clostridium_hungatei*, *Lactobacillus_reuteri*, and *Helicobacter_hepaticus* (Figure 7c). Compared to the DM group, the quantity of *Lactobacillus_reuteri* increased significantly in the PC, LXY, and HXY groups. In contrast, the relative abundance of *Clostridium_hungatei* and *Helicobacter_hepaticus* decreased significantly, restoring levels comparable to those in the CON group (Figure 7d).

## 4. Discussion

Xylose-oligosaccharide, a functional oligosaccharide, has demonstrated capabilities in regulating blood glucose, reducing blood lipid levels, and enhancing gut microbiota structure. As a prebiotic, xylose-oligosaccharide improves obesity and glucose intolerance induced by a high-fat diet and modulates plasma lipid profiles and intestinal microbiota in mice [24]. Additionally, xylose-oligosaccharide benefits intestinal mucosal barrier integrity and immune function [25]. This study revealed that xylose-oligosaccharide significantly alleviated insulin resistance, inhibited inflammatory responses, and mitigated intestinal microbiota disorders in T2D mice, thereby alleviating T2D symptoms in mice.

Two different doses of xylose-oligosaccharide were used to investigate their beneficial effects on T2D model mice. The hallmark of T2D mice is insulin resistance, accompanied by pancreatic islet dysfunction. Previous research has shown that functional sugars can improve insulin resistance. For instance, Li et al.’s study [26] found that tea polysaccharides possess hypoglycemic and hypolipidemic properties. In our study, the FBG of xylose-oligosaccharide-treated mice was significantly reduced. Furthermore, high-concentration xylose-oligosaccharide-treated diabetic mice exhibited a smaller increase in blood glucose after oral glucose administration, indicating improved glucose tolerance and insulin resistance. Pancreas tissue sections showed that both LXY and HXY groups displayed regeneration of impaired islets.

Diabetes is known to trigger inflammatory reactions and cause organ damage [27]. In our study, xylose-oligosaccharide treatment in diabetic mice downregulated inflammatory factors IL-1β, IL-6, and IL-10, thereby reducing inflammatory responses. xylose-oligosaccharide treatment also decreased the elevated MDA levels in diabetic mice, restoring them to the levels observed in the CON group, thus alleviating oxidative stress injury. Moreover, xylose-oligosaccharide treatment ameliorated pathological changes in kidney and liver tissues, restoring their functions to normal. These results suggested that xylose-oligosaccharide may alleviate T2D symptoms by reducing inflammation and oxidative stress, and mitigating damage to organs such as the kidney and liver.

The gut microbiota plays a fundamental role in obesity, diabetes, and other diseases, with diabetes being associated with significant biological disorders [28,29,30]. Some studies related to xylose have demonstrated its efficacy in regulating gut microbiota. For instance, xylan supplementation selectively promoted the growth of *Bifidobacterium pseudocatenulatum* in the pig intestine, alleviating intestinal dysbiosis caused by dietary fiber deficiency [31]. The research of Xiang et al. [32] explored the mechanism by which xylitol regulates intestinal homeostasis by promoting the growth of beneficial bacteria and the production of short-chain fatty acids. However, research primarily focuses on xylan, with limited studies on xylose-oligosaccharide. Xylose-oligosaccharides, with their smaller molecular structure, are more easily absorbed. This study showed that xylo-oligosaccharides can also regulate the balance of gut microbiota. Zhou et al.’s [33] research further revealed that adding dietary xylo-oligosaccharides to laying hens’ feed can increase the abundance of Lactobacillus, promote the production of certain short-chain fatty acids, and reduce the generation of pro-inflammatory metabolites such as lipopolysaccharides, thereby exerting prebiotic effects. The short-chain fatty acids exert their effects through two interconnected mechanisms: (1). Activation of the Nrf2 antioxidant pathway: by triggering the nuclear factor erythroid 2-related factor 2 signaling cascade, short-chain fatty acids induce the expression of antioxidant enzymes such as SOD and GSH-PX, thereby enhancing the scavenging of excessive reactive oxygen species and mitigating oxidative damage in tissues including the liver and pancreatic islets [34]. (2). Inhibition of the NF-κB inflammatory axis: acting via G protein-coupled receptors (GPR43/GPR109A), short-chain fatty acids promote the differentiation of regulatory T cells and suppress the nuclear factor kappa-light-chain-enhancer of activated B cells pathway, subsequently decreasing the production of pro-inflammatory cytokines such as TNF-α and IL-6 [35].

Through these integrated mechanisms, xylose-oligosaccharide-mediated gut microbiota regulation effectively modulates host oxidative stress and inflammatory responses in type 2 diabetes mouse models.

At the phylum level, an increased abundance of Firmicutes has been reported to elevate inflammatory cytokines and promote the inflammatory process in T2D [36]. Conversely, Bacteroidetes abundance has a negative association with T2D [37]. Our study found that xylose-oligosaccharide treatment increased the abundance of Bacteroidetes and Proteobacteria in diabetic mice while significantly decreasing the number of Firmicutes, all of which are significantly related to diabetes. At the genus level, xylose-oligosaccharide treatment restored *Lactobacillus_reuteri* levels and reduced *Clostridium_hungatei* and *Helicobacter_hepaticus* levels compared to T2D mice, mirroring the effects of melbine. Extensive research has proven the positive role of *Lactobacillus_reuteri* in intestinal microbiota and its therapeutic effect on diabetes [38,39,40]. T2D is associated with *Clostridium_difficile* infection, with elevated Clostridium content observed in diabetic patients [41]. Additionally, an increase in *Helicobacter_hepaticus* abundance has been noted in the gut microbiota of diabetic patients [42]. These findings support the role of xylose-oligosaccharide in alleviating gut microbiota dysbiosis, thereby improving T2D.

## 5. Conclusions

This study demonstrated that the dietary addition of xylose-oligosaccharide can prevent and treat T2D in model mice in a dose-dependent manner. It can directly reduce blood sugar levels and lipid accumulation in diabetic patients while also maintaining intestinal microbiota homeostasis. This study elucidated the mechanism underlying the association between xylose-oligosaccharide and intestinal microbiota, potentially providing a significant tool for further research on the prevention and treatment of metabolic syndromes such as diabetes.

## Data Availability

The original contributions presented in this study are included in the article. Further inquiries can be directed to the corresponding author.

## Abbreviations

The following abbreviations are used in this manuscript:

STZ	streptozotocin
CON	control group
DM	diabetic model group
PC	positive control group
LXY	low dose of xylose-oligosaccharide intragastric administration
HXY	high dose of xylose-oligosaccharide intragastric administration
OGTT	oral glucose tolerance test
ITT	insulin tolerance test
FBG	fasting blood glucose
TG	triglycerides
LDL-C	low-density lipoprotein cholesterol
HDL-C	high-density lipoprotein cholesterol
TCHO	total cholesterol
IL-1β	interleukin-1β
IL-6	interleukin-6
IL-10	interleukin-10
TNF-α	tumor necrosis factor-α
ELISA	enzyme-linked immunosorbent assay
SOD	superoxide dismutase
GSH	glutathione
GSH-PX	glutathione peroxidase
MDA	malondialdehyde
